# All-trans retinoic-acid inhibits heterodimeric bone morphogenetic protein 2/7-stimulated osteoclastogenesis, and resorption activity

**DOI:** 10.1186/s13578-018-0246-y

**Published:** 2018-08-23

**Authors:** Wenjuan Bi, Yi Liu, Jing Guo, Zhen Lin, Jinsong Liu, Miao Zhou, Daniel Wismeijer, Janak L. Pathak, Gang Wu

**Affiliations:** 1 School of Stomatology, North China University of Science and Technology, Tangshan, China; 2 Key Laboratory of Oral Medicine, Guangzhou Institute of Oral Disease, Affiliated Stomatology Hospital of Guangzhou Medical University, Guangzhou, China; 3 Department of Oral Implantology and Prosthetic Dentistry, Academic Centre of Dentistry Amsterdam (ACTA), University of Amsterdam and Vrije Universiteit Amsterdam, Amsterdam, The Netherlands; 4 Department of Orthopedics, The First Affiliated Hospital of Jinan University, Guangzhou, China; 5 School and Hospital of Stomatology, Wenzhou Medical University, Wenzhou, China

**Keywords:** Heterodimeric bone morphogenetic protein 2/7, All-trans retinoic acid, Osteoclastogenesis, Osteoclast activity, Bone loss, Bone regeneration

## Abstract

**Background:**

Bone regenerative heterodimeric bone morphogenetic protein 2/7 (BMP2/7) enhances but all-trans retinoic acid (ATRA) inhibits osteoclastogenesis. However, the effect of ATRA on physiological and/or BMP2/7-induced osteoclastogenesis in still unclear. In this study, we aimed to test the effect of combined treatment of BMP2/7 and ATRA on osteoclastogenesis, and resorption activity.

**Results:**

All-trans retinoic acid (1 µM) ± BMP2/7 (5 or 50 ng/ml) was added in murine pre-osteoclasts cell line RAW264.7 or mouse bone marrow derived macrophages (BMM) cultures. Osteoclast marker gene expression, osteoclastogenesis, and resorption activity were analyzed. BMP2/7 robustly enhanced osteoclast maker gene expression, osteoclastogenesis, and resorption activity. Interestingly, ATRA completely inhibited osteoclast formation in presence or absence of BMP2/7. Pan-antagonist of retinoic acid receptors (RARs) and antagonist of RARα, β or γ failed to reverse the inhibitory effect of ATRA on osteoclastogenesis. ATRA strongly inhibited *Rank* and *Nfatc1* expression.

**Conclusions:**

All-trans retinoic acid inhibits BMP2/7-induced osteoclastogenesis, and resorption activity possibly via RANKL–RANK pathway. Our findings from previous and current study suggest that combination of ATRA and BMP2/7 could be a novel approach to treat hyperactive osteoclast-induced bone loss such as in inflammation-induced severe osteoporosis and bone loss caused by cancer metastasis to bone.

**Electronic supplementary material:**

The online version of this article (10.1186/s13578-018-0246-y) contains supplementary material, which is available to authorized users.

## Background

Bone remolding is a tightly regulated process relying on balanced actions of bone-resorbing osteoclasts and bone-forming osteoblasts. A delicate regulation of this process is a prerequisite for normal bone homeostasis, and an imbalance is often linked to metabolic bone diseases, such as osteoporosis [1]. During osteoporosis, osteoclast activity is often elevated leading to excessive bone resorption. Systemic inflammatory diseases such as rheumatoid arthritis stimulate the formation and activity of osteoclasts, resulting excessive bone loss [2, 3]. Similarly, hyperactive osteoclast activity causes osteolysis and bone loss during cancer metastasis to bone [4]. Currently available bone anabolic therapeutic agent such as intermittent parathyroid therapy promotes bone regeneration via activation of osteoblast activity but continuous PTH therapy also increases osteoclast activity [5]. Bisphosphonate inhibits osteoclastogenesis but posses risk of adverse effects such as osteonecrosis of jaw [6]. Considering the fact that bone loss during systemic inflammation is caused by less osteoblast activity and high osteoclast activity, combined drug therapy that enhances osteoblast activity and inhibits osteoclast activity might be a smart approach.

Bone morphogenetic protein (BMPs) are members of TGF-β superfamily and have been reported in numerous studies as stimulator of osteogenesis [7]. BMPs bind to BMP receptor type I and II and initiate downstream signaling mainly SMADs [8]. BMPs signaling also occurs through SMAD-independent pathways via mitogen-activated protein kinases i.e., ERK, p38MAPKs, JNK [9, 10]. Genetic engineering allows the production of large amounts of BMPs for clinical use, and clinical trials have shown the benefits of FDA-approved recombinant human BMP2 and BMP7 homodimers [11]. BMP2/7 heterodimer not only has bone anabolic effect, but also inhibits breast cancer metastasis [12]. In our previous studies, we reported that BMP2/7 heterodimer at low-dose (5–50 ng/ml), in one hand, robustly enhances osteogenic differentiation compared to BMP2 or BMP7 homodimer alone [13], in other hand, enhances the RANKL-mediated osteoclastogenesis [14]. This double edge sword like property of BMP2/7 heterodimer is a serious concern about the use of it as a therapeutic agent for bone regeneration during osteoclast-induced bone loss. Therefore, a therapeutic agent that can reverse pro-osteoclastogenic effect of BMP2/7 without affecting osteoblast differentiation might address this concern.

All-trans retinoic acid (ATRA), an active metabolite of vitamin A, plays essential role in the embryological development of several tissue and organs [15]. Moreover ATRA is an important factor for skeletal development and metabolism [16–20]. In our previous study, we found that ATRA has a mild inhibitory effect on osteogenic differentiation of precursor cells and BMP2/7 partially reveres this effect [21]. Retinoic acid (RA) inhibits osteoclast formation via inhibition of NFATc1 and RANK [22–24]. Osteoclast inhibitory peptide 1 (OIP-1), also termed as a retinoic acid-induced gene expression, has been reported to inhibit osteoclast formation [25]. This suggests retinoic acid as a possible therapeutic agent to treat osteoporosis. If ATRA could reverse the stimulatory effect of BMP2/7 on osteoclastogenesis, and resorption activity, ATRA might be a novel drug to combine with BMP2/7 for bone regeneration in hyperactive osteoclast-induced bone loss. However, the effect of combination of ATRA and BMP2/7 on osteoclastogenesis, and resorption activity has not been investigated.

In this study, we hypothesized that ATRA inhibits physiological as well as BMP2/7-induced osteoclastogenesis. We cultured osteoclast precursors in presence of BMP2/7 and/or ATRA, and analyzed the effect of osteoclastogenesis, and resorption activity. Tartrate resistant acid phosphatase (ACP5), calcitonin receptor (CALCR), cathepsin K (CTSK), NFATc1, and RANK regulate osteoclastogenesis and resorption activity [26–29]. RANK–RANKL signaling is essential for osteoclastogenesis. RANKL binds to RANK and activates TNF receptor-associated factor 6 (TRAF6). TRAF6 and c-Fos pathways are the important downstream signaling of RANK-RANKL signaling. NFATc1 is a master switch for regulating terminal differentiation of osteoclasts, functioning downstream of RANKL [30]. Moreover, Coupling of c-Fos with NFAT family had been shown to be crucial for transcriptional events during osteoclastogenesis [31]. We analyzed the effect of BMP2/7 and/or ATRA treatment on *Acp5*, *Calcr*, *Ctsk*, *Nfatc1* and *Rank* gene expression. We also investigated the effect of treatments on macrophage markers *c*-*Fos*, and cell fusion marker *DC*-*Stamp* expression. ATRA is suggested to mediate the cellular effects via binding with nuclear retinoic acid receptors (RARα, β, γ) [32]. We investigated the possible role of RA–RARs signaling on anti-osteoclastogenic effect of ATRA.

## Results

### ATRA inhibited RAW264.7 cell proliferation

**Fig. 1:**
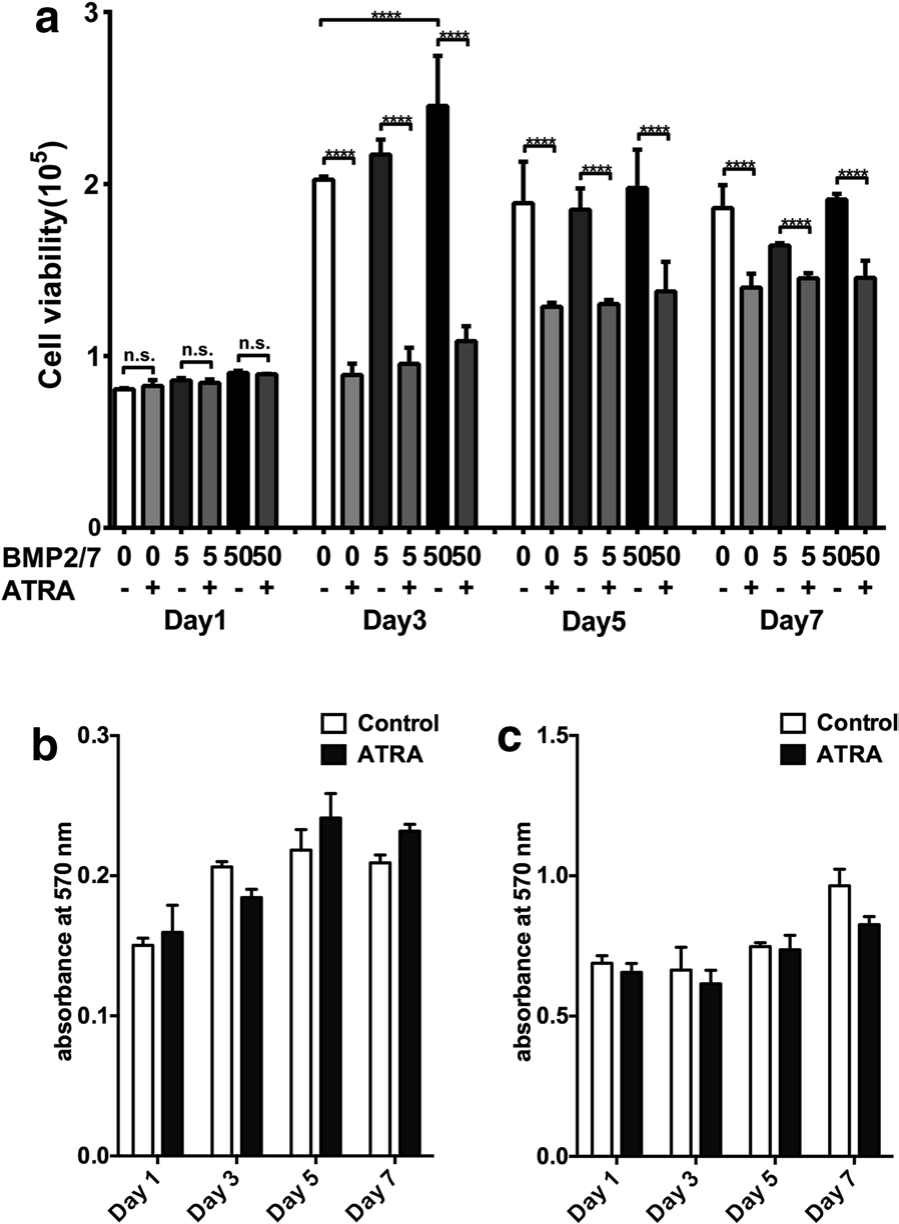
ATRA (1 µM) inhibited osteoclast precursor cells proliferation in presence or absence of BMP2/7 (5 or 50 ng/ml). Results of cell proliferation assay in RAW264.7 cell cultures (**a**). Cytotoxicity assay in BMM cell cultures (**b**), and RAW264.7 cell cultures (**c**). Values are mean ± SD, from three independent experiments. Significant effect of ATRA and/or BMP2/7 treatment, ****p < 0.0001, *n.s.* no significant difference

BMP2/7 and/or ATRA treatment did not affect the cell proliferation at day 1. BMP2/7 (50 ng/ml) treatment enhanced cell proliferation by 1.2-fold compared to control group at day 3, and ATRA reversed this effect. BMP2/7 (5 or 50 ng/ml) treatment did not affect cell proliferation at other time points. ATRA treatment reduced cell proliferation at day 3, 5 and 7 compared to control group (Fig. 1a). Cell proliferation was lower in ATRA + BMP2/7 (5 ng/ml), ATRA + BMP2/7 (50 ng/ml) groups compared to BMP2/7 (5 ng/ml) and BMP2/7 (50 ng/ml) group respectively (Fig. 1a). To rule out the cytotoxicity-caused inhibition of cell proliferation, we tested the cytotoxicity of ATRA. ATRA (1 µM) did not show cytotoxic effect on both RAW264.7 and BMM cell cultures at all the time points tested (Fig. 1b, c).

### ATRA treatment downregulated osteoclast marker gene expression in presence or absence of BMP2/7

**Fig. 2:**
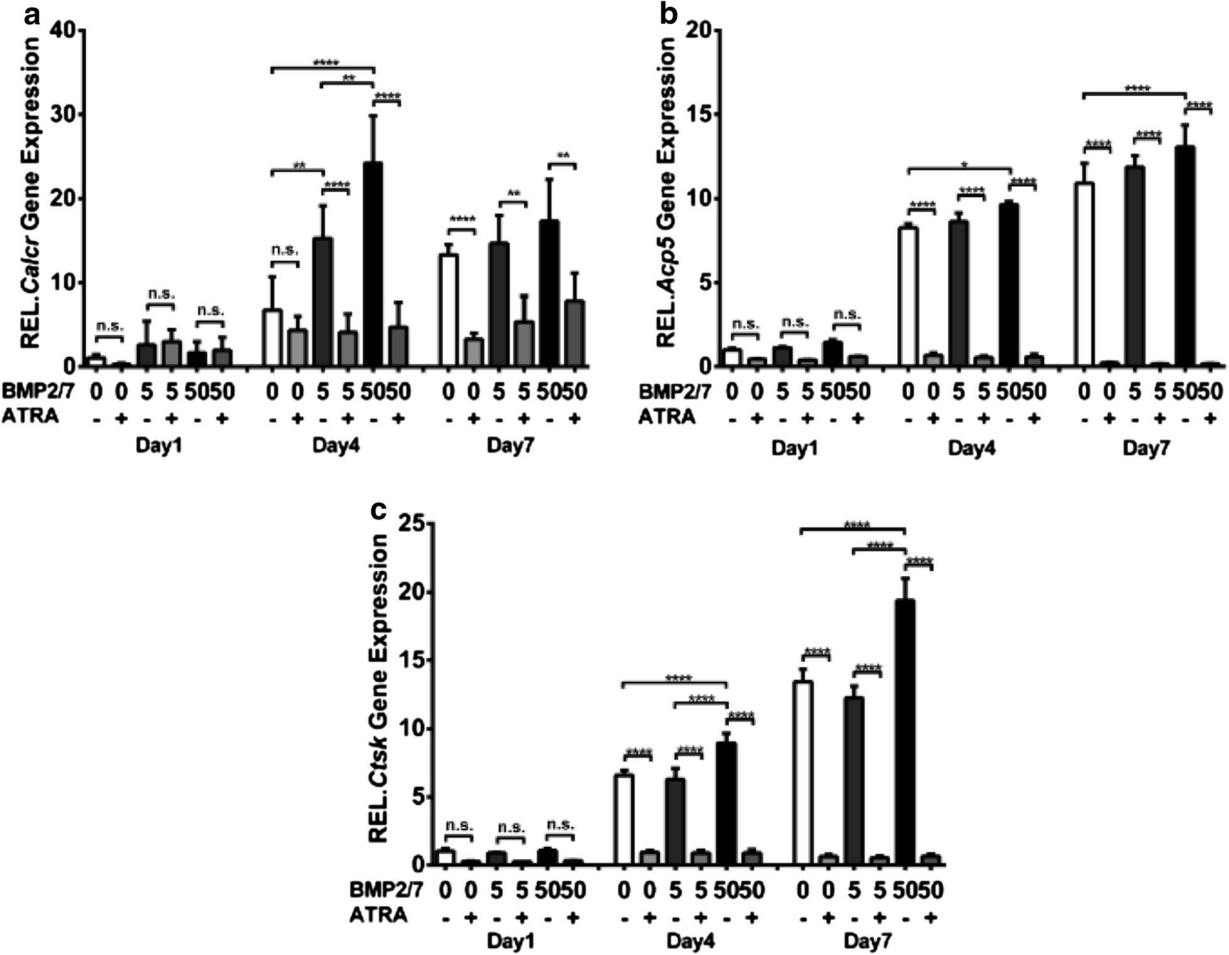
BMP2/7 (5 or 50 ng/ml) ± ATRA (1 µM) modulated osteoclast marker gene expression in RAW264.7 cell cultures. **a** *Calcr*, **b** *Acp5*, and **c** *Ctsk* expression in RAW264.7 cells at day 1, 4 and 7. Values are mean ± SD, from three independent experiments. Significant effect of the treatment *p < 0.05, **p < 0.01, ***p < 0.001, ****p < 0.0001, *n.s.* no significant difference

*Calcr*, *Acp5*, and *Ctsk* gene expression was upregulated in day 4 and 7 compared to day 1 in control group (Fig. 2a). BMP2/7 (5 or 50 ng/ml) upregulated *Calcr* gene expression compared to control group at day 4 (Fig. 2a). BMP2/7 (50 ng/ml) upregulated *Acp5* gene expression at day 4 and 7 compared to control group (Fig. 2b). BMP2/7 (50 ng/ml) upregulated *Ctsk* gene expression at day 4 and 7 compared to control and BMP2/7 (5 ng/ml) group (Fig. 2c). ATRA treatment did not affect *Calcr*, *Acp5*, and *Ctsk* gene expression (Fig. 2a–c) at day 1. ATRA treatment downregulated *Calcr*, *Acp5*, and *Ctsk* gene expression at day 4 and 7 compared to control and BMP2/7 (5 or 50 ng/ml) groups (Fig. 2a–c).

**Fig. 3:**
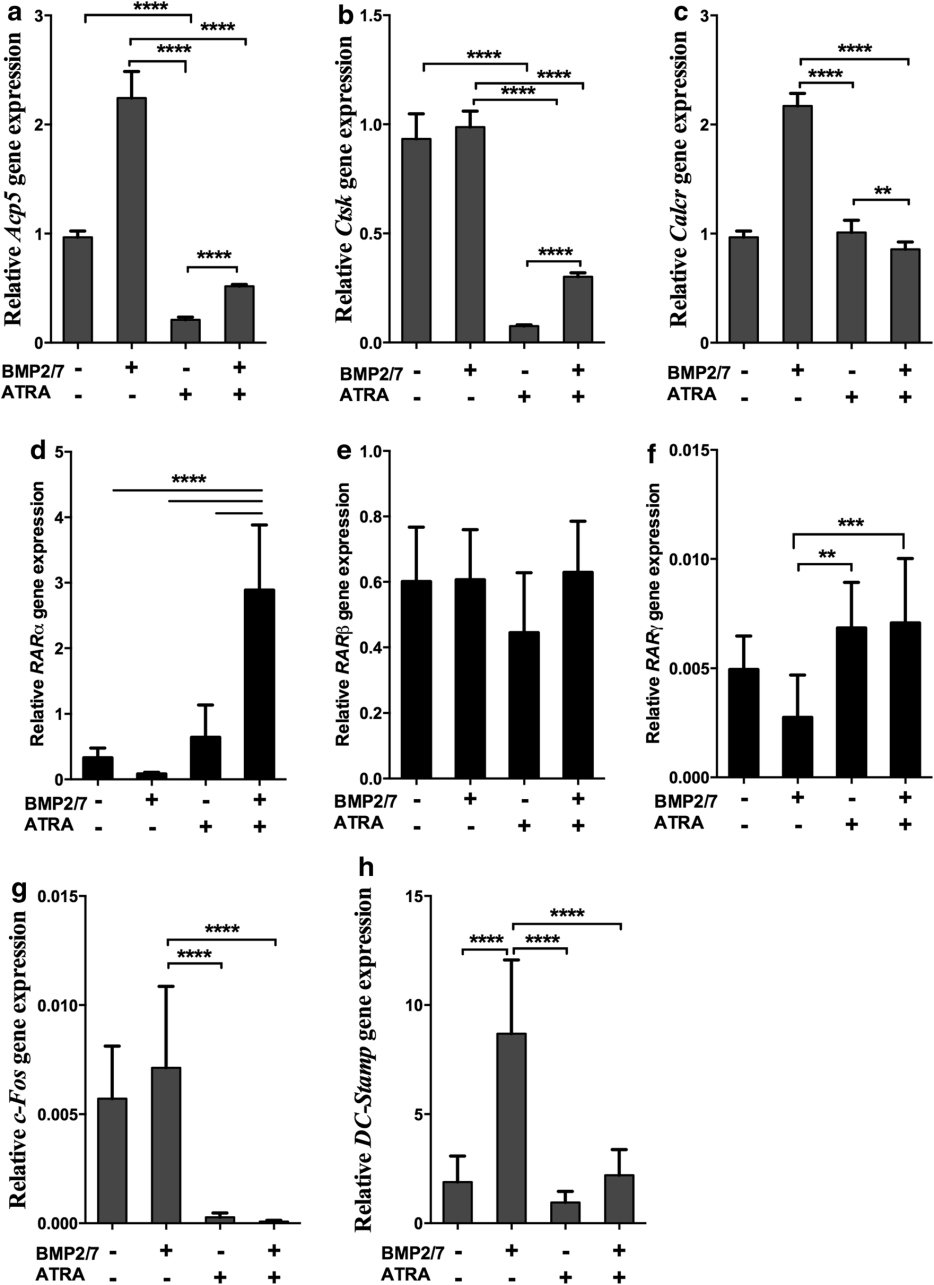
BMP2/7 (5 or 50 ng/ml) ± ATRA (1 µM) modulated osteoclast marker and RARs gene expression in BMM cell cultures (**a**–**c**). **d**–**f** RARs, **g** *C*-*fos* and **h** *DC*-*Stamp* expression in RAW264.7 cells. Values are mean ± SD, from three independent experiments. Significant effect of the treatment **p < 0.01, ***p < 0.001, ****p < 0.0001

We further tested the effect of ATRA on osteoclast marker gene expression in BMM cell cultures in presence or absence of BMP2/7. We found that BMP2/7 enhanced *Acp5* and *Calcr* gene expression and ATRA reversed this effect (Fig. 3a–c). BMP2/7 did not affect *Ctsk* gene expression, but ATRA inhibited *Ctsk* gene expression compared to control or BMP2/7 group (Fig. 3b). We found that *Rarα, Rarβ*, and *Rarγ* were expressed in RAW264.7 cells (Fig. 3d–f). ATRA + BMP2/7 treatment upregulated *Rarα* expression compared to in control, ATRA, and BMP2/7 groups (Fig. 3d). ATRA and/or BMP2/7 did not affect *Rarβ* expression (Fig. 3e). ATRA and ATRA + BMP2/7 group upregulated *Rarγ* expression compared to BMP 2/7 group (Fig. 3f). ATRA inhibited the expression of macrophage marker *c*-*Fos* in absence or presence of BMP2/7 (Fig. 3g). BMP2/7 did not affect the expression of *c*-*Fos* (Fig. 3g). BMP2/7 upregulated cell fusion marker *DC*-*Stamp* and ATRA reversed this effect (Fig. 3h).

### BMP2/7 treatment enhanced osteoclast formation and ATRA block osteoclast formation

**Fig. 4:**
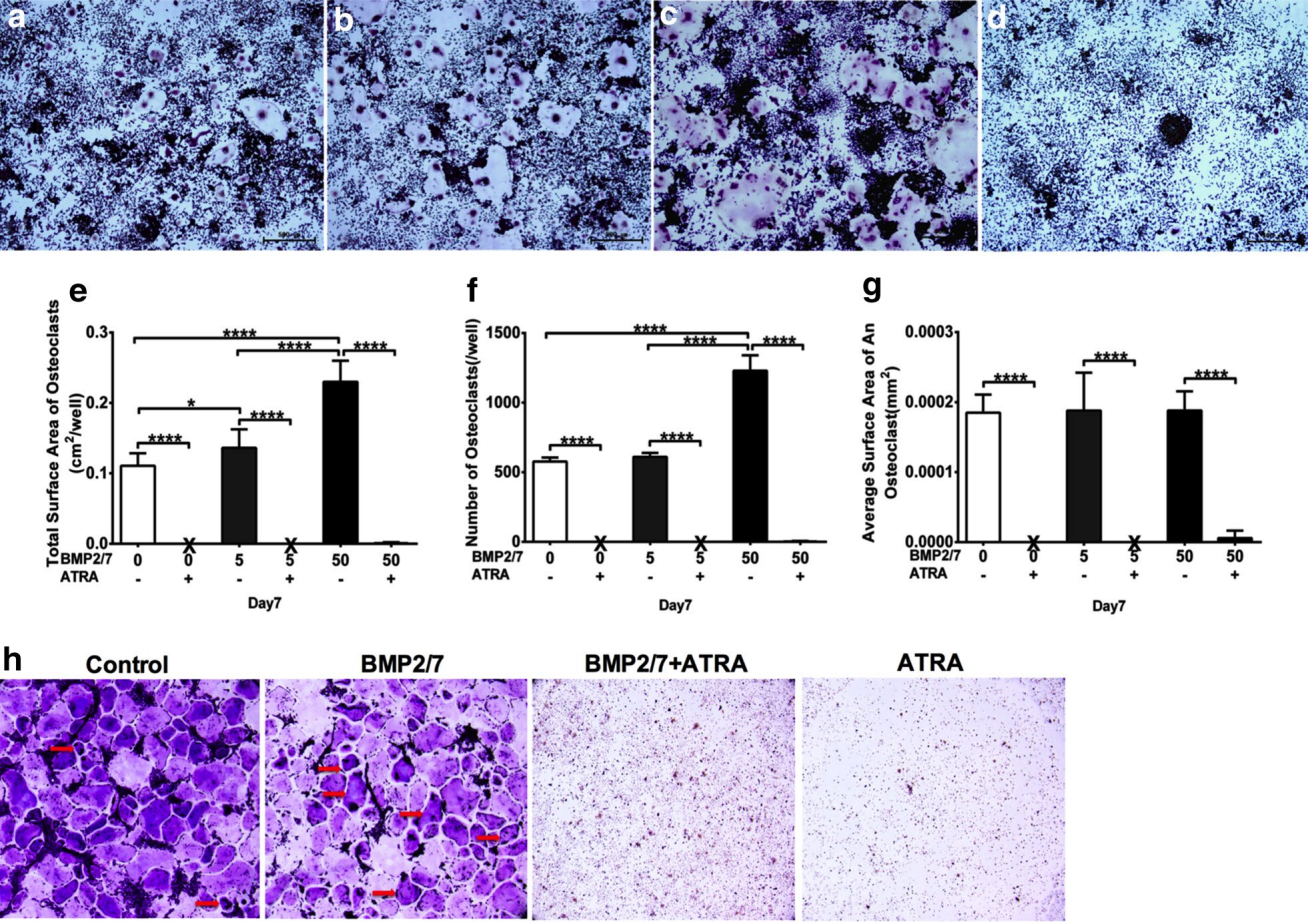
BMP2/7 (5 or 50 ng/ml) ± ATRA (1 µM) modulated osteoclast morphology. **a**–**d** Representative microscopic image of osteoclast morphology in RAW264.7 cell cultures: **a** control, **b** 5 ng/ml BMP2/7, **c** 50 ng/ml BMP2/7, and **d** 50 ng/ml BMP2/7 + ATRA group. **e**–**g** Semi-quantitative analysis of osteoclast morphology: **e** surface area of osteoclasts, **f** number of osteoclasts, and **g** average surface area of an osteoclast. Whitish circular structure with TRACP positive dark brown nuclei is osteoclast. X indicates nil. **h** Representative images of osteoclasts (multinucleated positive cells) formed in BMM cultures with or with out ATRA±BMP2/7 at day 7. All data are presented as mean ± SD, from three independent experiments. Significant effect of the treatment, *p < 0.05, ****p < 0.0001

In this study, osteoclast size varied approximately from Ø20 to Ø1500 μm. In BMP2/7 groups osteoclast size was Ø1000 μm. BMP2/7 5 ng/ml-group has more osteoclasts compared to control-group (Fig. 4a, b). More osteoclasts with higher TRACP intensity were observed in BMP2/7 50 ng/ml-group compared to control group and BMP2/7 5 ng/ml-group (Fig. 4a–c). In ATRA treated group osteoclasts were hardly observed (Fig. 4d). Osteoclasts total surface area was slightly higher in BMP2/7 5 ng/ml-group compared to control group (Fig. 4e). Osteoclast total surface area was 2- and 1.7-fold higher in BMP2/7 50 ng/ml-group compared to control and BMP2/7 5 ng/ml-group respectively (Fig. 4e). Number of osteoclasts in BMP2/7 50 ng/ml-group was twofold higher compared to control and BMP2/7 5 ng/ml-group respectively (Fig. 4f). BMP2/7 (5 or 50 ng/ml) treatment did not affect average surface area/osteoclast (Fig. 4g). Total surface area of osteoclasts, numbers of osteoclasts and average surface area/osteoclasts were almost nil in ATRA treated groups (Fig. 4e–g). We further tested the effect of ATRA (1 µM) and/or BMP2/7 (5 ng/ml) in osteoclastic differentiation of BMM. More numbers of osteoclasts were observed in BMP2/7 treated group and almost no osteoclasts were observed in ATRA treated group (Fig. 4h).

**Fig. 5:**
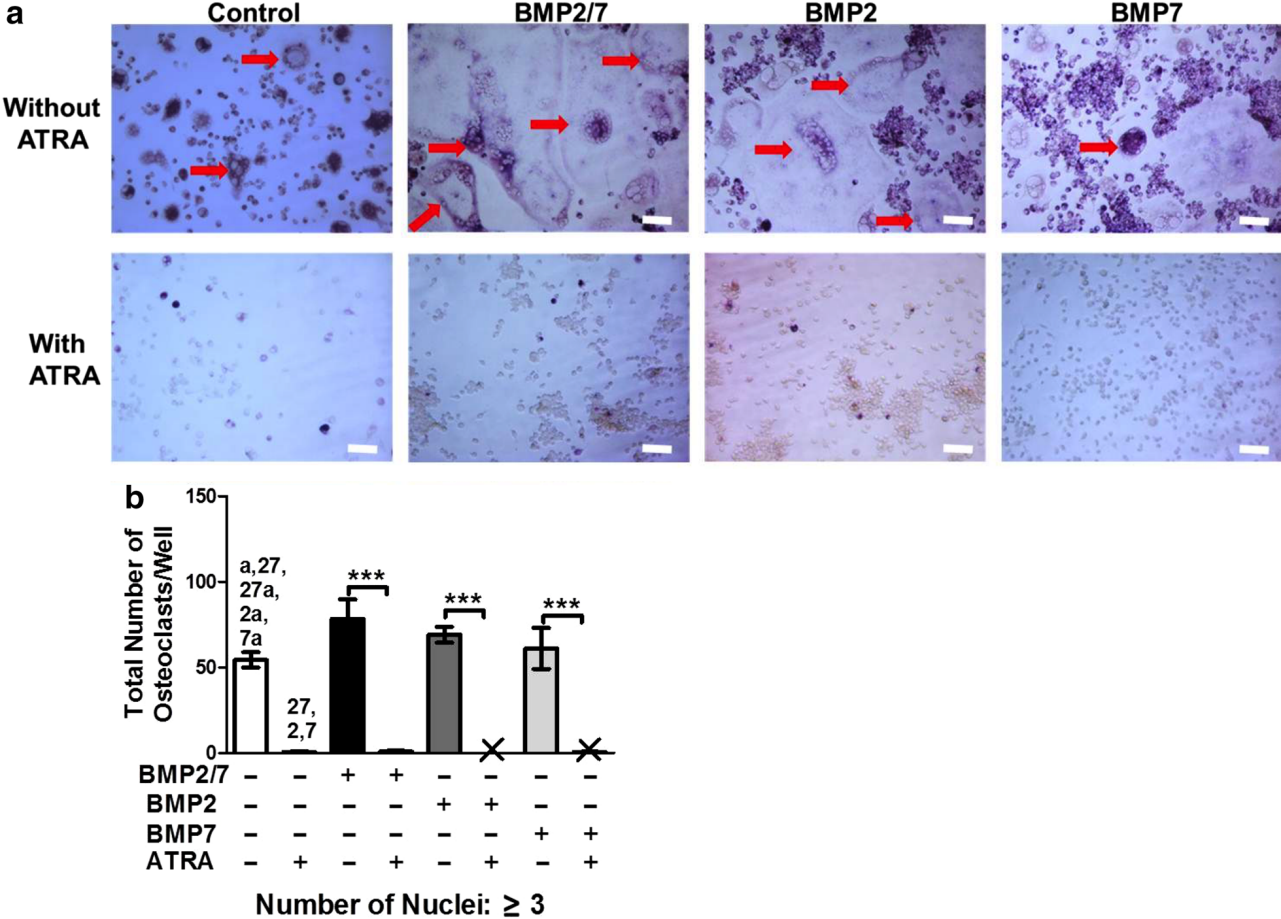
BMP2/7 (5 or 50 ng/ml) ± ATRA (1 µM) modulated osteoclastogenesis in RAW264.7 cell cultures. **a** Representative microscopic image of osteoclast formed in different groups: control, 5 ng/ml BMP2/7, 50 ng/ml BMP2/7, and 50 ng/ml BMP2/7 + 1 µM ATRA. Red arrow indicates the TRACP-positive multinucleated osteoclast. **b** Semi-quantitative analysis osteoclast numbers. X indicates nil. All data are presented as mean ± SD, from three independent experiments. Significant effect of the treatment, ****p < 0.0001, Significant difference also existed compared to the indicated group (p < 0.05): ^a^ no BMP2/7 + ATRA, ^27^ BMP2/7 + no ATRA, ^27a^ BMP2/7 + ATRA, ^2^ BMP2 + no ATRA, ^2a^ BMP2 + ATRA, ^7^ BMP7 + no ATRA, ^7a^ BMP7 + ATRA

BMP2 or BMP7 homodimer did not influence total osteoclastogenesis (Fig. 5a, b). BMP2/7 enhanced osteoclastogenesis by 1.4-fold compared to control group (Fig. 5b). Interestingly, ATRA was able to block osteoclastogenesis in presence of BMP2, BMP7 or BMP2/7 (Fig. 5a, b). Higher number of nuclei and surface area of osteoclasts indicates higher osteoclast activity. Number of osteoclasts with > 10 nuclei in BMP2/7-group was 5.5-, 2.3- and 3.6-fold higher compared to in control, BMP2 and BMP7-group respectively (Additional file 1: Figure S1). However, there was no difference in number of osteoclasts with 3–5 and 6–10 nuclei among these groups (Additional file 1: Figure S1).

### BMP2/7 enhanced and ATRA block osteoclast activity

**Fig. 6:**
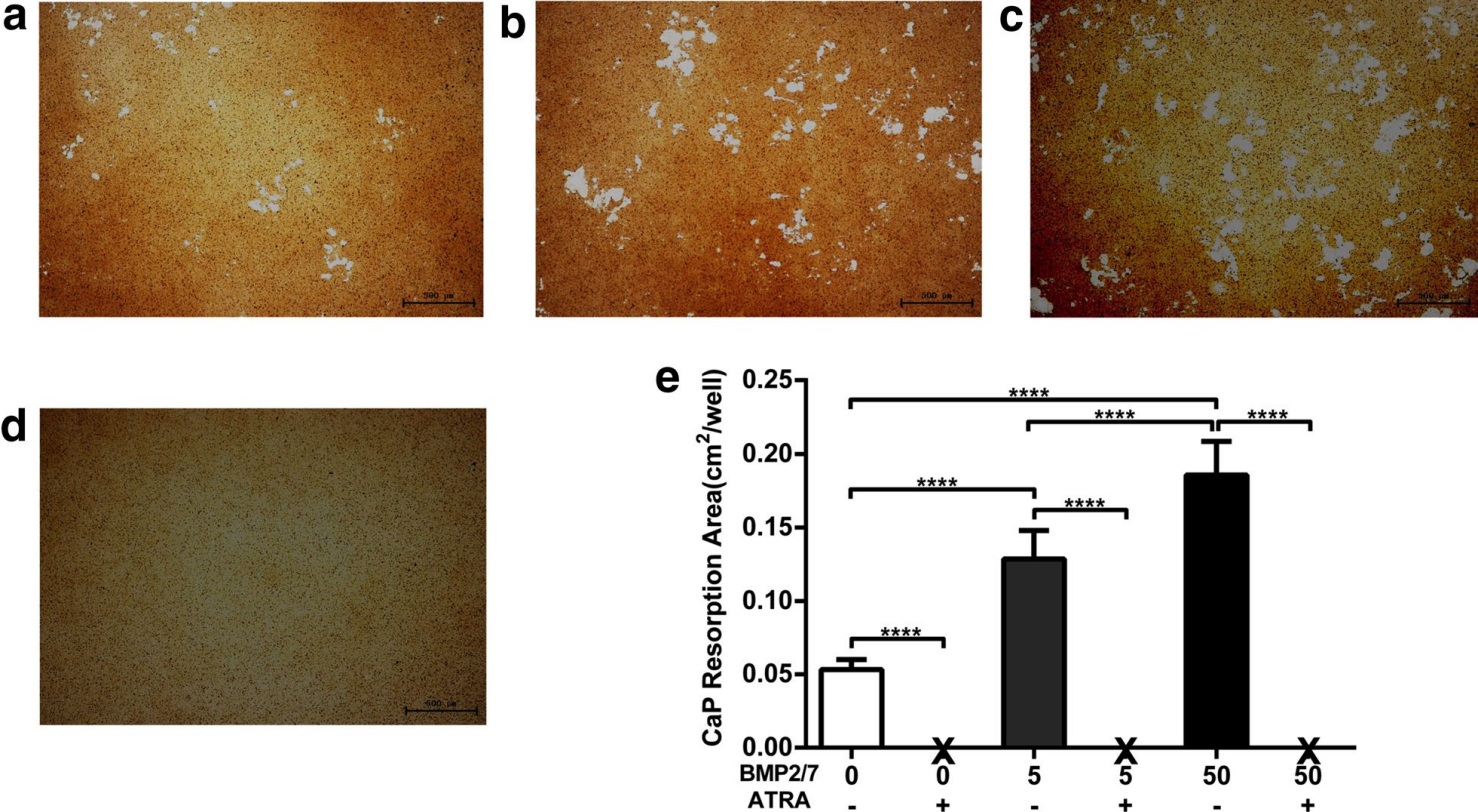
BMP2/7 (5 or 50 ng/ml) ± ATRA (1 µM) modulated osteoclast resorption activity. Representative microscopic image of calcium phosphate resorption by osteoclasts in different groups: **a** control, **b** 5 ng/ml BMP2/7, **c** 50 ng/ml BMP2/7, and **d** 50 ng/ml BMP2/7 + ATRA. **e** Semi-quantitative analysis of area of resorption pits. X indicates nil. All data are presented as mean ± SD, from three independent experiments. Significant effect of the treatment, ****p < 0.0001

More calcium phosphate resorption pits were visualized in BMP2/7 5 ng/ml-group compared to control group (Fig. 6a, b). Similarly, calcium phosphate resorption pits were more in BMP2/7 50 ng/ml-group compared to in control and BMP2/7 5 ng/ml-group (Fig. 6a–c). Calcium phosphate resorption pits were not observed in ATRA treated groups (Fig. 6d). Quantitative analysis of surface area of calcium phosphate resorption pits indicated that resorption pits surface area was 2.4-fold higher in BMP2/7 5 ng/ml-group compared to control-group (Fig. 6e). Moreover, in BMP2/7 50 ng/ml-group resorption pits area was 3.4- and 1.4-fold higher compared to in control and BMP2/7 5 ng/ml-group respectively (Fig. 6e), showing dose dependent effect of BMP2/7 on osteoclast activity. Resorption pit surface area was almost zero in ATRA treated group (Fig. 6e).

### Inhibitory effect of ATRA on osteoclastogenesis was not altered by RARs and pan-RAR antagonists

**Fig. 7:**
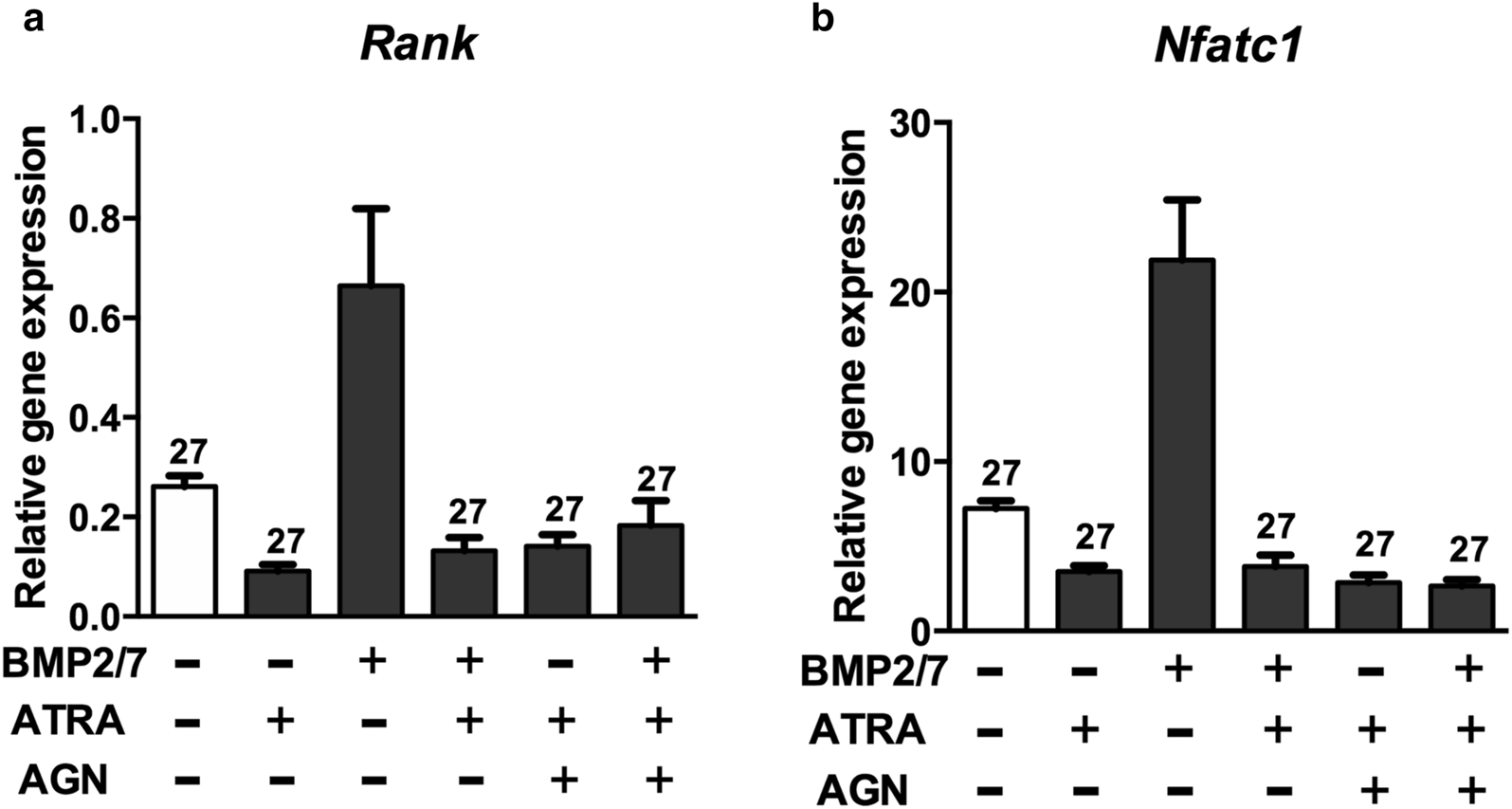
ATRA reversed BMP2/7-induced *Rank* and *Nfatc1* gene expression in osteoclast progenitors. **a** *Rank* and **b** *Nfatc1* gene expression. All data are presented as mean ± SD, from three independent experiments. Significant effect of the treatment compared to the indicated group: ^27^ BMP2/7. AGN: pan-RAR antagonist AGN

BMP2/7 (50 ng/ml) upregulated *Rank* and *Nfatc1* gene expression by ~3-fold compared to control group. ATRA treatment nullified this effect. The expression of *Rank* and *Nfatc1* gene was decreased in the presence of ATRA even with BMP2/7 and/or AGN (pan-RAR antagonist). AGN did not rescue the inhibitory effect of ATRA on *Rank* and *Nfatc1* gene expression (Fig. 7).

**Fig. 8:**
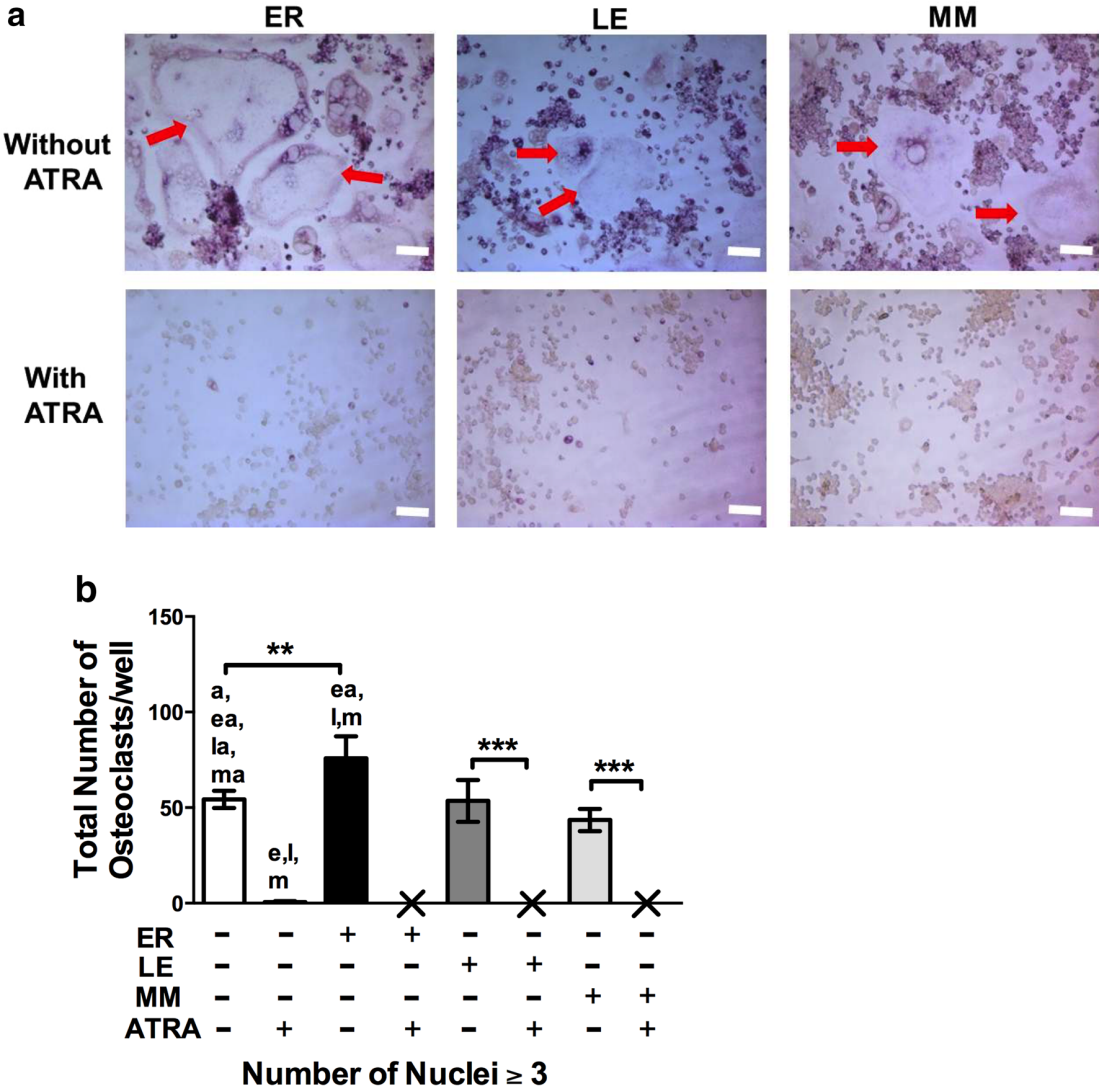
Individual RAR antagonist and pan-RAR antagonist were unable to rescue the inhibitory effect of ATRA on osteoclastogenesis. **a** Representative microscopic image of osteoclast formed in presence of BMP2/7 and/or ATRA ± 1.5 μΜ ER or 1.5 μΜ LE, or 1.5 μΜ MM at day 7. Red arrow indicates the TRAP-positive multinucleated osteoclast. **b** Semi-quantitative analysis of osteoclast numbers from the groups mentioned in (**a**). X indicates nil. All data are presented as mean ± SD, from three independent experiments. Significant effect of the treatment, **p < 0.01 and ****p < 0.0001. Significant difference also existed compared to the indicated group: ^a^ ATRA, ^e^ ER + no ATRAP, ^ea^ ER + ATRA, ^l^ LE + no ATRA, ^la^ LE + ATRA, ^m^ MM + no ATRA, ^ma^ MM + ATRA. ER: RARα-antagonist ER50891, LE: RARβ-antagonist LE135, and MM: RARγ-antagonist MM11253

Slightly more osteoclasts were observed in RARα-antagonist ER50891 treated group compared to the groups treated with other RAR antagonists (Fig. 8a). However, no osteoclast was observed when RARα-antagonist ER50891, RARβ-antagonist LE135, and RARγ-antagonist MM11253 were added in culture in combination with ATRA (Fig. 8a). Quantitative analysis of osteoclast numbers indicated that RARα-antagonist ER50891 stimulated osteoclast formation, but ER50891 was unable to rescue the ATRA-inhibited osteoclast formation (Fig. 8a, b). RARβ-antagonist LE135 or RARγ-antagonist MM11253 did not affect osteoclast formation when treated individually or in combination with ATRA (Fig. 8a, b). Numbers of osteoclasts with different numbers of nuclei (3–5, 6–10 and > 10) due to effect different RARs-antagonists ± ATRA are shown in Additional file 2: Figure S2.

## Discussion

BMP2/7 enhances osteogenic differentiation and bone formation, in contrast also stimulates osteoclastogenesis, and resorption activity. Therefore use of BMP2/7 as a bone anabolic agent to treat hyperactive osteoclastogenesis-caused bone degenerative diseases posses a risk. Combination of BMP2/7 with ATRA might eliminate such a risk since ATRA inhibits osteoclastogenesis, and resorption activity. However, the effect of BMP2/7 and ATRA in osteoclastogenesis, and resorption activity has not been investigated yet. In this study, we found that BMP2/7 enhanced osteoclastogenesis related gene expression, osteoclastogenesis, and osteoclast activity. ATRA alone or in combination with BMP2/7 downregulated osteoclastogenesis related gene expression and block the osteoclastogenesis, and resorption activity.

High dose BMP2 or BMP7 need to be administrated systemically to treat bone loss or osteoporosis, that posses a risk of systemic adverse effect [33–35]. We recently reported that a low concentration of BMP2/7 has a higher-potency on osteoblastogenesis compared to BMP2 or BMP7 homodimer alone [13]. In this study, we found that low-dose BMP2/7 enhanced expression of osteoclastogenesis related genes, osteoclastogenesis, and resorption activity that might accelerate bone loss instead of preventing. This finding is in accordance with the findings form our previous study [14]. ATRA has been reported to inhibit osteoclastogenesis, and resorption activity [22–24]. In this study, ATRA alone or in combination with BMP2/7 inhibited expression osteoclastogenesis related genes: *Rank, Nfatc1, Calcr, Acp5,* and *Ctsk*. Similarly ATRA inhibited macrophage marker *c*-*Fos* and cell fusion marker *DC*-*Stamp* in absence or presence of BMP2/7. This finding indicates the possible molecular mechanism of ATRA-inhibited osteoclastogenesis. Moreover, ATRA alone or in combination with BMP2/7 almost nullified osteoclastogenesis, and resorption activity. This is the first study to report the effect of the combination of ATRA and BMP2/7 on osteoclastogenesis, and resorption activity. Our findings provide insights on the possibility of using the combination of ATRA and BMP2/7 to treat hyperactive osteoclast-mediated bone loss.

In the absence of ATRA, the cell viability curve showed a bell-shaped, time-dependent tendency in the treatment of BMP2/7. The highest cell viability was 50 ng/ml BMP2/7 in day 3, and after 3 days treatment, there was no difference between control group and BMP2/7 groups. It is consist with our previous study, and showed that BMP2/7 has an earlier effect on osteoclast precursor proliferation [14]. However, in the presence of ATRA, the cell viability was significantly inhibited from day 3 to day 7, and BMP2/7 did not reverse the inhibitory effects of ATRA. Hu and colleagues reported that retinoic acid enhances human and murine osteoclast progenitors proliferation [36]. This discrepancy might be ATRA concentration related, i.e., Hu and colleagues used 4 nM and we used 1 μM ATRA. We found that ATRA inhibited BMP2/7-induced *RANK* expression in osteoclast precursors. Therefore, ATRA-mediated inhibition of BMP2/7-induced osteoclastogenesis might be via RANK-RANKL pathway. However, further in vitro and in vivo experimental verification are needed to prove this hypothesis.

BMP2/7 significantly enhanced CaP resorption this is in accordance with the findings from our previous study [14]. We found that BMP2/7 heterodimer enhanced osteoclast differentiation compared to BMP2 or BMP7 homodimer alone. Similarly, osteoclasts formed under influence of BMP2/7 were bigger in size and have more number of nuclei (> 10 nuclei) compared to control, BMP2 or BMP7 alone. More the number of nuclei and the surface area of the osteoclast, the bone resorbing activity will be the higher, this explains the higher CaP resorption in BMP2/7-group. Almost no CaP absorption was observed in BMP2/7 and/or ATRA treated groups, this seems obvious since in those group osteoclastogenesis was almost nil. Retinoic acid had been reported to induce osteogenic differentiation of mesenchymal stem cells [37]. Furthermore BMP2 and retinoic acid cooperate to induce osteogenic differentiation of preadipocytes [38]. Bisphosphonate is the commonly used drug to treat osteoporosis, however it poses the risk of adverse effects such as osteonecrosis and risk of atypical femur fracture [6]. Therefore, safer and more effective new anti-osteoporotic drugs are still in high demands. Based on the findings from literatures and our study, we can at least predict that low dose of ATRA might be able to nullify BMP2/7-induced osteoclastogenesis without attenuating osteogenesis.

In this study Pan-RAR-receptor antagonist, and receptor antagonist of RARα, RARβ or RARγ were unable to rescue the ATRA-mediated inhibitory effect on osteoclastogenesis. However, further osteoclastogenesis studies using RARs agonist and RARs ablated osteoclast precursors are needed to unravel the role of RA–RARs signaling in ATRA-mediated inhibition of osteoclastogenesis. Limitation of this study is that we used a high dose of ATRA compared to physiological dose. Hypervitaminosis A has been reported to play a role in bone loss and osteoporosis. Therefore, dose dependent effect of ATRA including physiological dose might provide important insights about using ATRA as an anti-osteoclastogenic agent. Bone regeneration is complex process in which both osteoblast and osteoclast formation, function and communication are connected to each other [39]. Therefore, the future studies focusing on the effect of combination of different doses of BMP2/7 and/or ATRA on osteoblast proliferation, differentiation and communication towards other bone cells are crucial. Similarly, the possible crosstalk/interaction between receptors of BMP2/7 and ATRA still need to be elucidated. Another limitation of this study is that we only tested the effect on BMP2/7 or/and ATRA on murine osteoclast precursors. Results from in vitro studies using murine cell line can not be translated in vivo, so further studies using primary human osteoclast precursors or in vivo studies focusing on effect of BMP2/7 and ATRA on osteoclastogenesis are recommended.

BMP2/7 not only enhances bone regeneration but also inhibits breast caner propagation and metastasis to bone [12]. During cancer and cancer metastasis to bone there is excessive bone loss due to hyperactive osteoclast activity. Therefore BMP2/7 alone might even induce the osteoclast-mediated bone loss. Combined BMP2/7 and ATRA treatment by using local drug delivery approach such as BMP2/7 and ATRA loaded nanoparticles targeting bone niche might be better approach to treat both metastasized cancer and metastasis-caused osteolysis [40]. Similarly a combination of BMP2/7 and ATRA might be used for implant fixation in excessive bone resorbed implantation area via surface modified implants that can load BMP2/7 and ATRA, and release locally [41]. Such approaches eliminate systemic adverse effect of BMP2/7 and ATRA, and give highest bone anabolic effect on the site of application.

## Conclusions

We reported that BMP2/7 enhanced osteoclastogenesis related gene expression, osteoclastogenesis, and resorption activity. ATRA alone or in combination with BMP2/7 downregulated osteoclastogenesis related gene expression and block the osteoclastogenesis, and resorption activity. Our findings from previous studies and current study suggest that the combination of a low dose of ATRA and BMP2/7 could be a novel therapeutic approach to treat hyperactive osteoclast-induced bone loss.

## Materials and methods

### Cell culture

Murine pre-osteoclast cell line (RAW264.7, ATCC, Cell Bank of Chinese Academy of Sciences) was cultured in Dulbecco’s minimum essential medium (DMEM, Gibco, Invitrogen) containing 10% fetal bovine serum (FBS, Gibco Invitrogen Corp., Grand Island, NY), and the medium was changed in every 3 days. Mice bone marrow-derived macrophages (BMM) were isolated from 6-week-old C57BL/6 male mice tibia and femur following the standard protocol described previously [42]. Cells were expanded and cultured in DMEM with 10% FBS. Cultures at ~ 80% confluency were trypsinized and used for different experiment described below. All animal experiments were approved by the Medical Ethics Committee of School of Stomatology, North China University of Science and Technology.

### Proliferation and cytotoxicity assay

RAW264.7 cells (2 × 10^4^/well) were seeded in 48-well plates. Cultures were treated with BMP2/7 (5 or 50 ng/ml, R&D Systems Inc., Minneapolis, MN) and rhRANKL (50 ng/ml) ± ATRA (1 μΜ, Sigma-Aldrich, St. Louis, MO, USA). In all the experiments, the medium and the drugs were changed in every 2 days. ATRA concentration was chosen based on the findings from our previous studies [21, 43]. Cell viability was determined at day 1, 3, 5 and 7 using alamar blue cell proliferation assay (Invitrogen Corporation, Carlsbad, CA, USA). The fluorescent intensity was measured using a fluorescence spectrometer (SpectraMax M5 Molecular Devices, Sunnyvale, CA, USA) at EX 540 nm/EM 590 nm.

Cytotoxicity assay was performed in RAW264.7 and BMM cell cultures by MTT assay. Cells (1 × 10^3^/well) were seeded in 96 well culture plates and treated with or without ATRA (1 μΜ) for 7 days. Then 20 μl of MTT solution (5 mg/ml) was added in each well. Cells were incubated with MTT solution for 4 h at 37 °C. The MTT solution was carefully removed, and 150 μl of dimethyl sulfoxide was added to each well. The absorbance was measured at 490 nm using an enzyme-linked immunosorbent assay (ELISA) microplate reader (Tecan i-control multiplate reader, Mannedorf, Switzerland).

### Real-time RT-PCR for osteoclast marker gene expression

RAW264.7 or BMM cells were seeded at concentration of 2 × 10^5^ cells/well in six-well plates. Cultures were treated with BMP2/7 (5 or 50 ng/ml) and rhRANKL (50 ng/ml) ± ATRA (1 μΜ) for 7 days. To analyze whether ATRA-mediated effect on osteoclast formation is via RARs activation, we added pan-RAR antagonist AGN (10 nM, apexbt 194310) in some cultures for 4 days. Total RNA was extracted using RNeasy Mini Kit and RNase-Free DNase Set (Qiagen GmbH, Hilden, Germany). Real-time-PCR was performed using PrimeScript ™ RT Reagent Kit, SYBR Premix ExTaq ™ (TaKaRa Biotechnology, Dalian, China), and ABI PRISM 7900HT Fast Real-Time PCR System with 384-Well Block Module (Applied Biosystems, Foster City, CA) to quantify the osteoclastic gene expression. We also quantified the expression of *Rarα, Rarβ*, and *Rarγ* in osteoclast progenitors. For quantitative real-time PCR, the values of relative target gene expression were normalized relative to housekeeping gene glyceraldehyde 3-phosphate dehydrogenase (*Gapdh*). The primers’ sequences (from Haojia Biotechnology Co., Ltd. Shanghai, China) used in this study are listed in Table 1.

**Table 1:** Primer pairs used for qPCR

Gene	Acc. no	Primer sequence	Size (bp)
*Ctsk*	NM_007802.3	F: 5′-CAGCAGAACGGAGGCATTGA-3′ R: 5′-CTTTGCCGTGGCGTTATACATACA-3′	84
*Acp5*	NM_001102405.1	F: 5′-CCAATGCCAAAGAGATCGCC -3′ R: 5′-TCTGTGCAGAGACGTTGCCAAG -3′	216
*Calcr*	NM_007588.2	F: 5′-TTACCGACGAGCAACGCCTAC-3′ R: 5′-AGCAAGTGGGTTTCTGCACTCA-3′	136
*Rank*	AF_019046.1	F: 5′-CACACCCAGGACTATCATCAT-3′ R: 5′-CCCACCAAAGCATCTTCTGA-3′	133
*Nfatc1*	EU_887572.1	F: 5′-TGGTGGCTTACCTTTCCCAA-3′ R: 5′-GCTCTTCACAGTCGTGCGAA-3′	81
*Rarα*	NM_001177302	F: 5′-AGCAGGCTCTACCTTGCCCT-3′ R: 5′-CAGTGGAAACCCAGCAGGAA-3′	84
*Rarβ*	NM_001042727	F: 5′-AAGTTAGTCTGCCGTCTGGAC-3′ R: 5′-TTGCCCATACCTTCAAGCAT-3′	99
*Rarγ*	NM_001042727	F: 5′-TGTGTTCATCCCTGTCCTGT-3′ R: 5′-CGAAAGGCAGTGCTGAGATT-3′	128
*c*-*Fos*	NM_010234.2	F: 5′-TGAGCAGTCAGAGAAGGCAA-3′ R: 5′-TTCACGAACAGGTAAGGTCCT-3′	133
*DC*-*Stamp*	NM_001347395.1	F: 5′-CCTGCTGCTCACAGATGGT-3′ R: 5′-TGGTGGTTGATGTTGGGAT-3′	128
*Gapdh*	NM_001289726.1	F: 5′-ACCACAGTCCATGCCATCAC-3′ R: 5′-TCCACCACCCTGTTGCTGTA-3′	452

*Ctsk*: cathepsin K, *Acp5*: acid phosphatase 5 tartrate resistant, *Calcr*: calcitonin receptor, *Nfatc1*: nuclear factor of activated T-cells cytoplasmic 1, Rar: retinoic acid receptor, *Gapdh*: aldehyde-3-phosphate dehydrogenase

### Osteoclastogenesis

RAW264.7 or BMM cells were seeded at concentration of 1.5 × 10^4^ cells/well in 96-well plates. Cultures were treated by BMP2/7 heterodimer, BMP2 or BMP7 homodimer (50 ng/ml), rhRANKL ± ATRA (1 μΜ). To test the role of individual RAR on ATRA-mediated inhibition of osteoclast formation, we added the RARα-antagonist ER50891 (1.5 μΜ, Cat. No. 3823, TOCRIS.), RARβ-antagonist LE135 (1.5 μΜ, Cat. No. 2021, TOCRIS.) or RARγ-antagonist MM11253 (1.5 μΜ, Cat. No. 3822, TOCRIS.) in some RAW264.7 cell cultures. After 7 days of culture, the cells were processed to TRACP staining (TRACP kit, Sigma-Aldrich). TRACP positive (brown–red staining) multinucleated cells (≥ 3 nuclei) were regarded as osteoclasts. Applying a random sampling protocol, the number of TRACP positive multinucleated cells were counted using a combination of light and fluorescence microscopy (Leica DFC320; Leica Microsystems, Wetzlar, Germany) at a final magnification of 100×. Cells were categorized into 1 of the following groups based on number of nuclei: osteoclasts with ≥ 3 nuclei, 3–5 nuclei, 6–10 nuclei and > 10 nuclei. Total surface area of osteoclasts and surface area of an osteoclast were also analyzed.

### Resorption activity assay

Osteoclast resorption activity was performed using hydroxyapatite substrate coated culture plates. Cells were seeded at a concentration of 4 × 10^4^ cells/well in 24-well Osteoclast Activity Assay Substrate plates (OAAS, OCT USA, Inc., Irvine, CA). Cultures were treated with BMP2/7 (5 or 50 ng/ml) and rhRANKL (50 ng/ml) ± ATRA (1 μΜ) for 7 days. Cultures were washed with 6% sodium hypochlorite solution to remove the cells. The wells were examined in a Nikon microscope with NIS-Elements F2.20 and photographed in color at a final magnification of 100×. Approximately 24 photomicrographs were collected per well using a systematic random-sampling strategy. The photomicrographs were printed in color for histomorphometric analysis. The resorption areas that exhibited white color on the pale brown background were measured using the point-counting technique [44].

### Statistical analysis

Each experiment was performed 3 times, and each time in triplicates. All the data are presented as mean ± SD. Comparisons among the groups were made by one-way analysis of variance (ANOVA). Post Hoc comparisons were made using Bonferroni corrections. The level of significance was set at p < 0.05. SPSS software (version 20) for a Windows computer system was employed for the statistical analysis.

## Additional files

**Additional file 1: Figure S1.** Semi-quantitative analysis of osteoclast numbers based on number of nuclei in different groups from Fig. 5a: 50 ng/ml BMP2/7, 50 ng/ml BMP2, 50 ng/ml BMP7 in presence or absence of 1 µM ATRA. All data are presented as mean ± SD, from 3 independent experiments, n = 9. Significant effect of the treatment, ****p<0.001, Significant difference also existed compared to the indicated group: ^a^ no BMP2/7 + ATRA, ^27^ BMP2/7 + no ATRA, ^27a^ BMP2/7 + ATRA, ^2^ BMP2 + no ATRA, ^2a^ BMP2 + ATRA, ^7^ BMP7 + no ATRA, ^7a^ BMP7 + ATRA.

**Additional file 2: Figure S2.** Semi-quantitative analysis of osteoclast numbers from the groups mentioned in Fig. 8a based on number of nuclei. All data are presented as mean ± SD, from 3 independent experiments, n = 9. Significant effect of the treatment, ****p<0.001, Significant difference also existed in compared to indicated group: ^a^ ATRA, ^e^ ER + no ATRAP, ^ea^ ER + ATRA, ^l^ LE + no ATRA, ^la^ LE + ATRA, ^m^ MM + no ATRA, ^ma^ MM + ATRA. ER: RARα-antagonist ER50891, LE: RARβ-antagonist LE135, and MM: RARγ- antagonist MM11253.
