# Supplementary figures and images for: All-trans retinoic-acid inhibits heterodimeric bone morphogenetic protein 2/7-stimulated osteoclastogenesis, and resorption activity
Source: Cell Biosci. 2018 Aug 23;8:48. doi: 10.1186/s13578-018-0246-y (PMC6107948; doi:10.1186/s13578-018-0246-y)

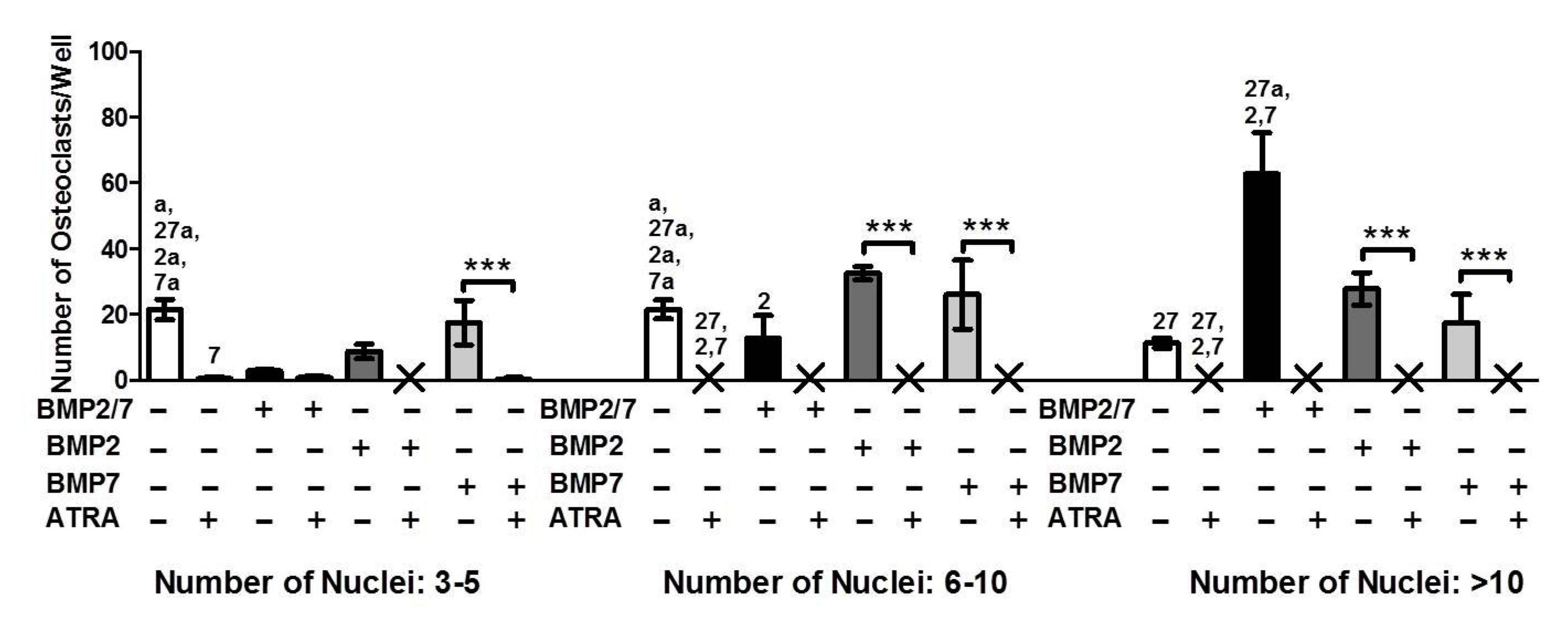

Supplement: Supplementary file 1 — Additional file 1: Figure S1. Semi-quantitative analysis of osteoclast numbers based on number of nuclei in different groups from Fig. 5a: 50 ng/ml BMP2/7, 50 ng/ml BMP2, 50 ng/ml BMP7 in presence or absence of 1 µM ATRA. All data are presented as mean ± SD, from 3 independent experiments, n = 9. Significant effect of the treatment, ****p<0.001, Significant difference also existed compared to the indicated group: a no BMP2/7 + ATRA, 27 BMP2/7 + no ATRA, 27a BMP2/7 + ATRA, 2 BMP2 + no ATRA, 2a BMP2 + ATRA, 7 BMP7 + no ATRA, 7a BMP7 + ATRA. [file 13578_2018_246_MOESM1_ESM.tiff]

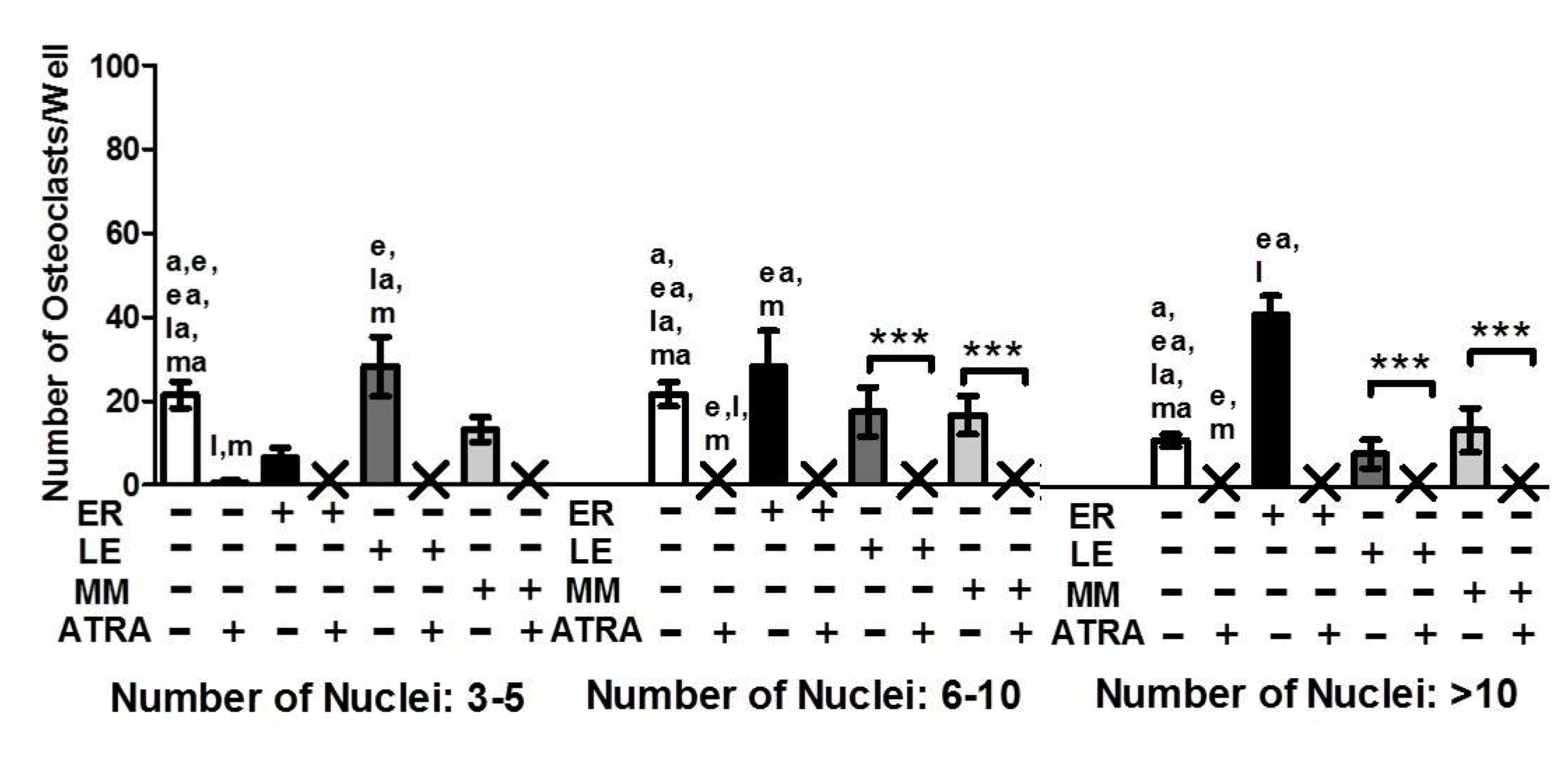

Supplement: Supplementary file 2 — Additional file 2: Figure S2. Semi-quantitative analysis of osteoclast numbers from the groups mentioned in Fig. 8a based on number of nuclei. All data are presented as mean ± SD, from 3 independent experiments, n = 9. Significant effect of the treatment, ****p<0.001, Significant difference also existed in compared to indicated group: a ATRA, e ER + no ATRAP, ea ER + ATRA, l LE + no ATRA, la LE + ATRA, m MM + no ATRA, ma MM + ATRA. ER: RARα-antagonist ER50891, LE: RARβ-antagonist LE135, and MM: RARγ- antagonist MM11253. [file 13578_2018_246_MOESM2_ESM.tiff]
